# Supplementary material for: Educational Strategies to Reduce Physician Shortages in Underserved Areas: A Systematic Review
Source: Int J Environ Res Public Health. 2023 May 29;20(11):5983. doi: 10.3390/ijerph20115983 (PMC10252282; doi:10.3390/ijerph20115983)
Supplement: Supplementary file 1 [file ijerph-20-05983-s001.zip › ijerph-2344281-supplementary.pdf]

Table S1– Summary of studies selected for qualitative evaluation.

Authors/Year	Description of the context and characteristics of the educational experience	Objectives	Methodology	Study results	Score (MERSQI)
Rabinowitz et al.,1999 [19]	The Jefferson Medical School (JMC) Physician Shortage Area Program (PSAP) selects tickets from rural areas and small towns that manifest a commitment to act as doctors in similar areas. The PSAP presents specific curricular activities that emphasize medical practice in rural areas.	To evaluate whether the PSAP graduates remain in rural areas in the long term (retention for 22 years).	Cohort study that compares student graduates from PSAP (n:206) and JMC students who did not participate in PSAP (n:2793). Descriptive analysis with data from 1978 to 1981 graduates. U.S.A.	JMC's Rural Program (PSAP) graduates represent 12% of all FM physicians in rural Pennsylvania. Overall, PSAP graduates were much more likely than their JMC non-PSAP colleagues to practice in a rural area of the United States (34% vs 11%; RR, 3.0), to practice in a scarcity area (30% vs 9%; RR, 3.2), to practice family medicine (52% vs 13%; RR, 4.0), and to practice family medicine in a rural area (21% vs 2%; RR, 8.5).	13.2
Rabinowitz et al., 2005 [17]	The Jefferson Medical School (JMC) Physician Shortage Area Program (PSAP) selects tickets from rural areas and small towns that manifest a commitment to act as doctors in similar areas. The PSAP presents specific curricular activities that emphasize medical practice in rural areas.	To evaluate whether the PSAP graduates remain in rural areas in the long term (retention for 11 to 16 years).	Cohort study that compares student graduates from PSAP (n:148) and JMC students who did not participate in PSAP (n:1789). Analysis performed using Pearson's test with data from 1997 to 1986 graduates. United States of America (U.S.A.).	After 11–16 years, 68% (26/38) of PSAP graduates still practiced family medicine in the same rural area, compared to 46% (25/54) of their non-PSAP peers (p = 0.03). Survival analysis showed that PSAP graduates practice FM in the same rural area for longer than non-PSAP graduates (p = 0.04).	15.6
Pacheco et al., 2005 [33]	The University of New Mexico develops a residency program with different practice scenarios (urban X rural).	To assess whether different practical training scenarios influence the decision to practice in rural areas.	A cross-sectional study that compares graduates that carried out their activities in rural settings (n:63) and the group with activities in urban areas (n:89). The analysis used a Chi square test with data related to graduates from 1978 to 2005. U.S.A.	In total, 65.1% of the graduates of residences in rural environments were practicing in rural area, while the percentage of the residents wanting to carry out training in the city of Albuquerque and that practiced in rural areas was 25.8% (p<0.001)	14.4

Worley et al., 2008 [28]	The University of Flinders Medical School develops three clinical training programs in the third year of the course: The first in rural primary care services (PRCC), the second in remote area tertiary hospital (NTCS), and the third in urban tertiary hospital (Flinders Medical Center, FMC).	To analyze whether the students' formative itinerary influences their decision to choose a degree in general medicine.	Cross-sectional study comparing graduates of PRCC (n:13), NTCS (n:16) and FMC (n:45). The analysis used a Chi square test with data from 1978 to 1986 graduates. Australia.	The percentage of students who completed the third year of graduation in rural areas and chose the Family Medicine (FM) specialty was 62%. This percentage was 53% in the group that was in a tertiary hospital in a remote area and 38% in the group of the usual model at the central campus. However, there was no significant difference between these groups ($p = 0.067$; $p = 0.24$).	10
Halaas et al., 2008 [29]	The Rural Physician Associate Program (RPAP) is an experience of clinical immersion in rural communities associated with preceptorship and curricular activities focused on clinical practice in rural areas.	To analyze whether RPAP increases the choice of performing Family Medicine (FM) as a specialty.	A cohort study that compares the percentage of graduates from RPAP (n:901) who chose FM with the average percentage of other students from the same school and the national average. Descriptive analysis with data related to graduates from 1978 to 1986. U.S.A.	The percentage of students who chose FM among RPAP graduates was almost 70% in 1996 and dropped to only 60% in 2006. The percentage of UMN graduates who did not participate in RPAP and chose FM was 19.9% in 1996 and 7.9% in 2006. In the U.S.A., the percentage of graduates that choose FM was below 20% in 1996 and dropped to less than 10% in 2006.	13.2
Longombe, 2009 [31]	The first private college in a rural area was created in 1989 while there were 10 private colleges in urban areas in the People's Republic of Congo.	To evaluate if the graduates of this rural school are more likely to practice in rural areas after graduation when compared to a private school implemented at a similar time.	Cohort study with two groups: graduates from rural university (n:43) and graduates from an urban school (n:107). The statistical analysis was performed with a T test and a Chi square test with data related to graduates from 2001 to 2010. People's Republic of Congo.	A total of 81.4% of the graduates of the rural school were working in rural areas and 26.7% graduated from the urban school (X2 = 38.41, df = 1, $p<0.01$).	13.8
Brokaw et al., 2009 [30]	The University of Indiana carries out training activities in the initial years at its main campus and at regional campuses. Later stages of training take place on the main campus with the same format for all students.	To evaluate whether conducting the first two years of medical school in the regional campus influences the choice to practice FCM.	Cohort study comparing students from regional campuses (n:1200) and students with all training at the Indianapolis Campus (n:1211). Multiple logistic regression analysis with data from 1974 to 1994. U.S.A.	Students who attended the Evansville, South Bend, Muncie or Terre Haute campuses in the early years of the course were significantly more likely (ORs = 2.43-1.74) to practice as FM physician, compared	13.2

				to students who completed the entire training on the Indianapolis campus.	
Ferguson et al., 2009 [32]	The University of Massachusetts Medical School (UMMS) has a residency program with 3 different outpatient settings: Community Health Center (in a poor urban area), an outpatient clinic linked to a hospital 30 miles away from the city, and an outpatient clinic linked to a tertiary hospital in the urban area.	To assess whether the performance of clinical practices in different settings in the medical residence influence the choice of clinical practice in an area of scarcity at the beginning of the career and at the time of the study (2009).	Cross-sectional study in which graduates of the group of residents of the Community Health Center (n:82) were compared to a control group practicing in a hospital in an urban area (n:93). The analysis used logistic regression with data related to the graduates from 1997 to 2004. U.S.A.	The doctors who performed their outpatient training in the Community Center were more likely to start their careers (OR = 5.61; 95% CI = 2.01-15.7) and work in areas of scarcity (OR = 4.53; 95% CI = 1.43-14.35) when compared with residents who performed their training in an urban hospital.	11
Matsumoto et al., 2010 [24]	The Jichi Medical University (JMU) Medical Training Program recruits and subsidizes students from all regions of Japan. The training at this university is linked to a curriculum with an emphasis on clinical practice in rural areas and is associated with a nine-year contract to provide services in rural areas.	To assess whether JMU graduates continue to practice in rural areas after the end of the mandatory nine-year contract.	Cross-sectional study with two groups: students from Jichi University (n:484) and a control (n: 270,371). Descriptive analysis with data from 1998 to 2000 graduates. Japan.	The percentage of doctors who graduated from Jichi Medical University, practicing in regions with a lower population density, is 8.7%, while 1.1% of doctors in Japan work in regions with similar characteristics.	13.2
Rabinowitz et al., 2012 [22]	Programs developed to expand the medical workforce in rural areas in 3 different states of the U.S.A. These programs select students from rural areas and offer curricular activities focused on medical performance in rural areas.	To analyze whether the graduates of these programs have greater professional insertion in rural areas and if they choose FM as a specialty.	A cohort study that compares the graduates of these Rural Programs (n:1754) and doctors trained abroad who practice in these 3 states (n:6474). The analysis uses a T test with data related to graduates from 1978 to 1991.	In total, 63.8% of the doctors trained in the Rural Programs of 3 institutions (located in Pennsylvania, Minnesota and Illinois) were practicing in rural areas, while 26.5% of the doctors trained abroad were working in rural areas. The group of students who participated in the Rural Programs presented a greater chance of practicing FM in rural areas when compared to doctors who were trained abroad (RR 10.0;8.7–11.6; $p <$ 0.01).	16.8
Rabinowitz et al., 2013 [18]	The Jefferson Medical School (JMC) Physician Shortage Area Program (PSAP) selects tickets from rural areas and small towns that manifest a commitment to act as doctors in similar areas. The PSAP	To evaluate whether PSAP graduates remain in rural areas in the long term	A cohort study that compares students who egressed from the PSAP who began their careers practicing FM in rural areas (n:37) and JMC students who did not participate in the PSAP	Of the 37 PSAP graduates who originally began their careers practicing FM in rural areas, 26 (70.3%) continued in the same rural area in 2011. Among the non-	15.6

	presents specific curricular activities that emphasize medical practice in rural areas.	(retention for 20 to 25 years).	(n:52). The analysis was performed using Pearson's test with data related to graduates from 1974 to 2004. U.S.A.	participants of the PSAP, 52 physicians who began their careers practicing as FM physicians in rural areas, 24 (46.2%) remained in the same rural area (*p*= 0.02).	
MacDowell et al., 2013 [20]	The University of Illinois develops a program (Rural Medical Education—RMED) with curricular activities and an admissions process aimed at training doctors to work in rural areas.	To analyze whether University of Illinois graduates who have participated in RMED have a greater chance of choosing FM or practice in rural areas when compared to their colleagues who have not participated in the program.	A cohort study comparing RMED graduates (n:160) and university graduates that did not participate in RMED (n:2663). The analysis used a Chi square test with data from 1972 to 2009 graduates. U.S.A.	RMED graduates were more likely (OR: 14.38; 10.16-20.35) than non-graduates of RMED to choose FM as a specialty and more likely (OR:17.20; 12.18-24.35) to practice in a rural setting.	15.6
Crump et al., 2013 [26]	The University of Louisville offers medical training on a small campus to expand the chance to train doctors to work in rural areas.	To compare graduates from the training program in Trover municipality and their colleague graduates from the University of Louisville.	A cohort study comparing students from the Campus of Trover (n:57) and the University of Louisville (n:1294). The analysis used a Chi square test with data regarding graduates from 1988 to 1997. U.S.A.	Graduates from the Trover Campus were more than four and a half times more likely to choose an FM residency than graduates who remained in Louisville during the clinical cycle (OR 4.50; CI 3.06-6.63; <.001). Graduates from the Trover Campus were six times more likely to practice in rural areas or small townships (6.27; CI 4.26–9.24; <.001).	15.6
Shelker et al., 2014 [23]	The University of Otago's medical course has 2 approaches to train doctors to work in rural areas: an admission process that emphasizes the rural origin and a program for the insertion of students in rural areas throughout the fifth year of the course.	To evaluate the influence of the admission of students from rural areas and/or insertion for one year in a rural setting during the clinical cycle on the choice of FM as a specialty after graduation.	A cohort study comparing students who participated in these interventions (n:112) and students who did not (n:621). The analysis was performed using a Chi square test. New Zealand.	Graduates from the program with specific admission for students of rural origin and/or who were inserted in rural areas during their training were more likely to choose postgraduate training at a rural hospital or choose FM as a specialty (OR 2 .1, 95%CI 1.2–3.6, *p*<0.01).	13.2

Sen Gupta et al., 2014 [27]	James Cook University has the mission to train doctors to work in rural areas and has developed strategies for inserting training in rural areas.	To assess whether the inclusion of students in rural areas in the undergraduate program influences the choice of an FM specialty.	A cohort study that compares graduates who attended boarding school in a rural area (n:108) and a control group who attended boarding school in an urban area (n:134). Analysis using Pearson's test with data related to graduates from 1971 to 2006. Australia.	Students from urban areas who had clinical internships in non-urban areas had a greater chance of working in rural areas (OR: 6.1; CI2.9-12.6; $p<0.01$) when compared to students from urban areas who had clinical internships only in urban areas.	14.4
Fuglestad et al., 2017 [21]	The Duluth Medical School (DMS) of the University of Minnesota conducts a selection process that seeks students with potential to work in rural areas and a curriculum with an emphasis on generalist training.	To analyze if graduates of the Duluth Medical School choose to specialize in FM and/or practice in a rural area, and the characteristics related to these choices.	Cohort study comparing the percentage of Duluth campus graduates (in:1773) that choose FM, and the average U.S. percentage. Descriptive analysis with data on graduates from 2005 to 2011.	In total, 47% of DMS graduates (n=837) began their careers as FM physicians, while the national average is about 10%.	13.2
Techakehakij et al., 2017 [25]	The Collaborative Project to Increase the Production of Rural Physicians (CPIRD) recruits students from rural areas of Thailand. CPIRD students carry out their preclinical cycle together with the other students. However, they carry out their clinical cycle training in centers mostly outside the big cities and near their regions of origin. At the end of their studies, students of the CPIRD's public university and the usual training circuit must work for 3 years in rural public hospitals.	To analyze the influence of the CPIRD in the retention of doctors in rural hospitals after the 3 year mandatory public contract.	A cohort study that compares graduates from the CPIRD (n:4766) and graduates formed in the traditional model (n:10487). Analysis through multiple logistic regression with data related to graduates from 2008 to 2011. Thailand.	Students who have participated in the CPIRD program have a greater chance of staying in hospitals in rural areas after 3 years (OR 2.441; 2.192-2.719).	15.6
